# Effect of the Combination of Everolimus and Mesenchymal Stromal Cells on Regulatory T Cells Levels and in a Liver Transplant Rejection Model in Rats

**DOI:** 10.3389/fimmu.2022.877953

**Published:** 2022-06-10

**Authors:** Morgan Vandermeulen, Pauline Erpicum, Noella Bletard, Laurence Poma, François Jouret, Olivier Detry

**Affiliations:** ^1^ Department of Abdominal Surgery and Transplantation, University of Liege Hospital [Centre Hospitalier Universitaire (CHU) ULiege], Liege, Belgium; ^2^ Centre de Recherche et de Développement du Département de Chirurgie (CREDEC), Groupe Interdisciplinaire de Génoprotéomique Appliquée (GIGA), Cardiovascular Sciences, University of Liege (ULiege), Liege, Belgium; ^3^ Laboratory of Translational Research in Nephrology (LTRN), Groupe Interdisciplinaire de Génoprotéomique Appliquée (GIGA), Cardiovascular Sciences, University of Liege (ULiege), Liege, Belgium; ^4^ Division of Nephrology, University of Liege Hospital [Centre Hospitalier Universitaire (CHU) ULiege], Liege, Belgium; ^5^ Department of Pathology, University of Liege Hospital [Centre Hospitalier Universitaire (CHU) ULiege], Liege, Belgium

**Keywords:** experimental model, cell-therapy, stem cells, mTOR inhibitors, graft tolerance

## Abstract

**Introduction:**

Mesenchymal stromal cells (MSCs) have particular properties that are of interest in organ transplantation, including the expansion of regulatory T cells (Tregs), a key factor in transplant tolerance induction. However, the most effective immunosuppressive drug to associate with MSCs has yet to be defined. Additionally, the impact of the association of everolimus with MSCs on Treg expansion, and on the induction of liver graft tolerance, has never been studied. The aim of this study was to evaluate the effects of MSCs in combination, or not, with everolimus on Treg expansion and in a model of rejection after liver transplantation (LT) in the rat.

**Methods:**

Firstly, 24 Lewis rats were assigned to 4 groups (n=6 in each group) receiving intravenous MSCs or saline injection at day (D)9 with/without subcutaneous everolimus from D0 to D14. Analysis of circulating Tregs was performed at D0, D14 and D28. In a second set of experiment, 30 Lewis rats were randomized in 3 groups 48hours after LT with a Dark Agouti rat liver: everolimus (subcutaneous for 14 days), MSCs (intravenous injection at post-operative day 2 and 9), or both everolimus and MSCs. Rejection of the liver graft was assessed by liver tests, histology and survival.

**Results:**

Individually, MSC infusion and everolimus promoted Treg expansion in rats, and everolimus had no negative impact on Treg expansion in combination with MSCs. However, in the LT model, injections of MSCs two and nine days following LT were not effective at preventing acute rejection, and the combination of MSCs with everolimus failed to show any synergistic effect when compared to everolimus alone.

**Conclusion:**

Everolimus may be used in association with MSCs. However, in our model of LT in the rat, post-transplant MSC injections did not prevent acute rejection, and the association of MSCs with everolimus did not show any synergistic effect.

## 1 Introduction

Mesenchymal stromal cells (MSCs) are a heterogeneous population of fibroblast-like cells which have been defined by the International Society for Cellular Therapy (1). They can be isolated from various tissues, including bone marrow (2, 3). MSCs have been used in many fields, including solid organ transplantation (SOT), in which they are considered to be a promising cell therapy. *In vitro* and *in vivo* experiments have demonstrated that MSCs have interesting immunomodulatory properties (4–6), combined with beneficial effects on ischemia-reperfusion injury and on tissue/organ repair (7). One of the most favorable effects of MSCs in SOT could be their capacity of inducing the expansion of CD4^+^ CD25^+^ FoxP3^+^ regulatory T cells (Tregs) (6, 8). Indeed, increased levels of circulating Tregs in peripheral blood have been identified as a key factor in inducing transplant tolerance after SOT (9). Still, despite encouraging pre-clinical data, preliminary clinical studies using MSC therapy in SOT patients have shown inconsistent results, including after liver transplantation (LT) (4, 10, 11). The immunosuppressive drugs (ISDs), necessarily combined with MSC infusion, probably explain in part the controversial observations of MSC therapy in SOT (10). *In vitro*, high doses of calcineurin inhibitors (CNIs) have been shown to be toxic for MSCs and to antagonize their immunomodulatory properties (12). *In vivo*, CNI effects on MSCs have been variable, sometimes promoting, sometimes antagonizing MSC function (13), while Treg function and proliferation have been inhibited by CNIs both *in vitro* and *in vivo* (14, 15). In a recent clinical trial our group failed to find any difference in Treg levels in LT recipients receiving MSCs when compared to a control group (16). Amongst the other available ISDs that are clinically used in SOT, mammalian/mechanistic target of rapamycin (mTOR) inhibitors*, i.e.* rapamycin (sirolimus) and everolimus, inhibit mTOR complexes. This leads to the blockade of T-cell progression in response to growth factors or cytokines, thereby limiting T-cell proliferation (17). *In vitro*, rapamycin inhibits MSC proliferation and antagonizes its inhibitory effect (18). However, in contrast to what has been observed *in vitro*, the *in vivo* combination of MSCs and rapamycin has been shown to promote graft tolerance in cardiac or islet allograft models (19, 20). However, such an immunosuppressive association has never been tested in a LT model.

The “ideal” ISD to be associated with MSCs in SOT has not yet been defined, and the impact of everolimus in association with MSCs on Treg expansion and on the establishment of liver graft tolerance has never previously been studied. Given that both MSCs and everolimus appear to have a positive impact on Tregs, their association deserves to be investigated. The aim of this study was to evaluate the effects of MSC injections in combination, or not, with everolimus, on Treg expansion and to test the hypothesis that this association prevents acute rejection in a LT model in the rat.

## 2 Material and Methods

All experimental procedures were approved by the Animal Ethics Committee of the University of Liege, Belgium (Protocol number #1957). All animals received humane care according to the criteria outlined in the “Guide for the Care and Use of Laboratory Animals” prepared by the National Academy of Sciences and published by the National Institutes of Health (NIH publication 86-23 revised 1985). The authors followed the institutional and national guidelines for the care and use of laboratory animals. All rats were purchased from Janvier Labs (Le Genest-Saint-Isle, France). All invasive procedures were performed under anesthetic gas (isoflurane), and prevention of post-operative pain was achieved with a dose of buprenorphine (0.05 mg/kg) at the end of each procedure. Rats had access to water and food *ad libitum* throughout the duration of the experiment.

### 2.1 Everolimus Administration

Everolimus (E-4040, LC Laboratories, MA) was administered through a subcutaneous (sc) osmotic pump (#2002, Alzet, Cupertino, CA). It had previously been dissolved in a mixture containing 50% dimethyl sulfoxide (Sigma-Aldrich, MO), 40% propylene glycol (Fagron, Rotterdam, The Netherlands) and 10% absolute ethanol (VWR, PA), to obtain a solution with an everolimus concentration of 4.17mg/mL. 250µL of this solution was then used to fill each osmotic pump. After sc implantation the pump delivered the solution at a constant flow of 0.5µL/h for 14 days, as defined by the manufacturer’s specifications, corresponding to a dose of everolimus of 0.25mg/kg/day. In the groups not receiving everolimus, the pumps were filled with the same mixture but without the addition of everolimus (placebo). To implant the pump a shaved and washed site posterior to the scapula was incised, and a sc pocket was created with the aid of a hemostatic clamp before insertion of the filled pump. The wound was then closed with sutures.

### 2.2 Isolation, *In Vitro* Expansion and Identification of MSCs

The protocol of isolation, culture and identification of bone marrow-derived MSCs from Lewis rats was performed according to our previously reported protocol (21). In brief, bone marrow was collected by flushing femurs and tibias of 10-week-old inbred Lewis rats. The bone marrow was then homogenized in Phosphate-Buffered Saline (PBS, Lonza) + 2% fetal bovine serum (FBS, Lonza) before being filtered and centrifuged twice. Cells were cultured in 75cm^2^ culture flask (Falcon) with DMEM supplemented with 10% FBS, 1% penicillin (Lonza) and 1% L-Glutamin (Lonza). Cells were cryopreserved before the third passage. When needed, cells from 3 donors were thawed in a water-bath at 37°C, centrifuged and re-suspended together in DMEM culture medium. MSC were cultured at 37°C in a humidified 5%CO_2_ incubator. Supplemented DMEM was changed twice a week. Cells were split in two culture flasks when they reached 80% of confluency. Phenotyping was performed twice: before cryopreservation and before i.v. injection. The cells were adherent to plastic and presented fibroblast-like morphology. MSC positive markers were evidenced by flow-cytometry, using APC-conjugated anti-rat CD90 (BD Pharmingen) antibody and AlexaFluor-conjugated anti-rat CD29 antibody (BD Pharmingen). MSC were negative for V450-conjugated anti-rat CD45 (BD Horizon) antibody, MSC were negative for PE-conjugated anti-CD79a (abcam) antibody and for FITC-conjugated anti-rat CD11b (BD Pharmingen) antibody. The ability of MSC to differentiate into adipocytes, osteoblasts and chondroblasts lineages was demonstrated by positive staining for Oil Red O, Alizarin Red, and toluidine blue, respectively. In addition, the differentiation status into adipogenic (FABP4/DAPI), chondrogenic (Aggrecan/DAPI) and osteogenic (Osteocalcin/DAPI) lineages by immunofluorescence was confirmed using a commercial kit (R&D systems, #SC020, USA) (**Supplementary Figure 1**).

### 2.3 MSC Administration

Trypsin - Ethylenediaminetetraacetic acid (EDTA) was used to detach MSCs from the culture flasks. Cells were then centrifuged in DMEM cell culture media at 500G for 5 minutes (min) and counted using a Thoma chamber. MSC were used only before the fifth passage. The required number of cells was resuspended in 1ml of pre-warmed saline and slowly injected through the penile vein of the rat. Rats not treated with MSCs received an equal volume of pre-warmed saline.

### 2.4 Experimental Design

**Figure 1:**
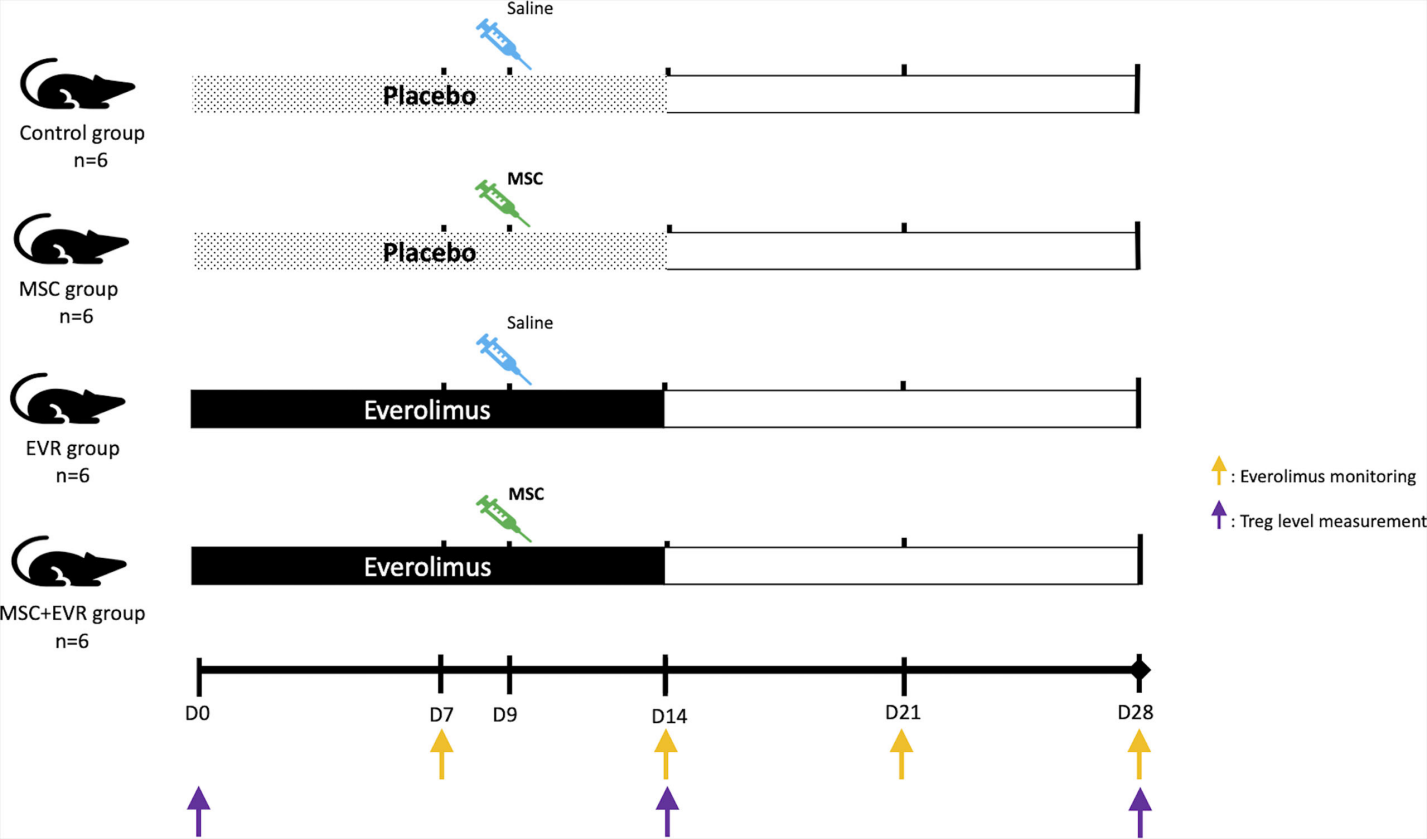
Evaluation of the effect of MSCs and everolimus on Treg proliferation (experimental design). Pumps were implanted at D0 and functioned for 14 days. One injection of MSCs or saline was performed at D9. Everolimus blood levels were monitored once a week (orange arrows); Treg blood levels were measured by flow cytometry on D0, D14 and D28 (purple arrows). D, day; EVR, everolimus; MSC, mesenchymal stromal cell; Treg, regulatory T cells.

The first part of the experiment aimed at studying the efficiency of MSCs and of the combination of MSCs with everolimus in promoting Tregs. Twenty-four 7-week-old male Lewis rats were randomly assigned to 4 groups (**Figure 1**): *MSC+EVR group* (n=6), received the association of sc everolimus from D0 to D14 and intravenous (iv) MSC injection (3.10^6^cells/kg) at D9; *MSC group* (n=6), received placebo administration sc from D0 to D14 and iv MSC injection (3.10^6^cells/kg) at D9; *EVR group* (n=6), received everolimus sc from D0 to D14 and iv saline injection at D9; *Control group* (n=6), received placebo sc from D0 to D14, and iv saline injection at D9.

A fluorescence-activated cell sorting (FACS) analysis of circulating Tregs was performed at D0, D14 and D28. 250µL of whole blood was obtained in an EDTA tube (BD Microtainer K2E tubes, 365975) at D7, D14, D21 and D28 post-pump implantation to monitor everolimus blood levels (**Figure 1**). Samples were analyzed using an UHPLC-MS/MS Acquity^®^ analyzer (Waters, #186002350).

**Figure 2:**
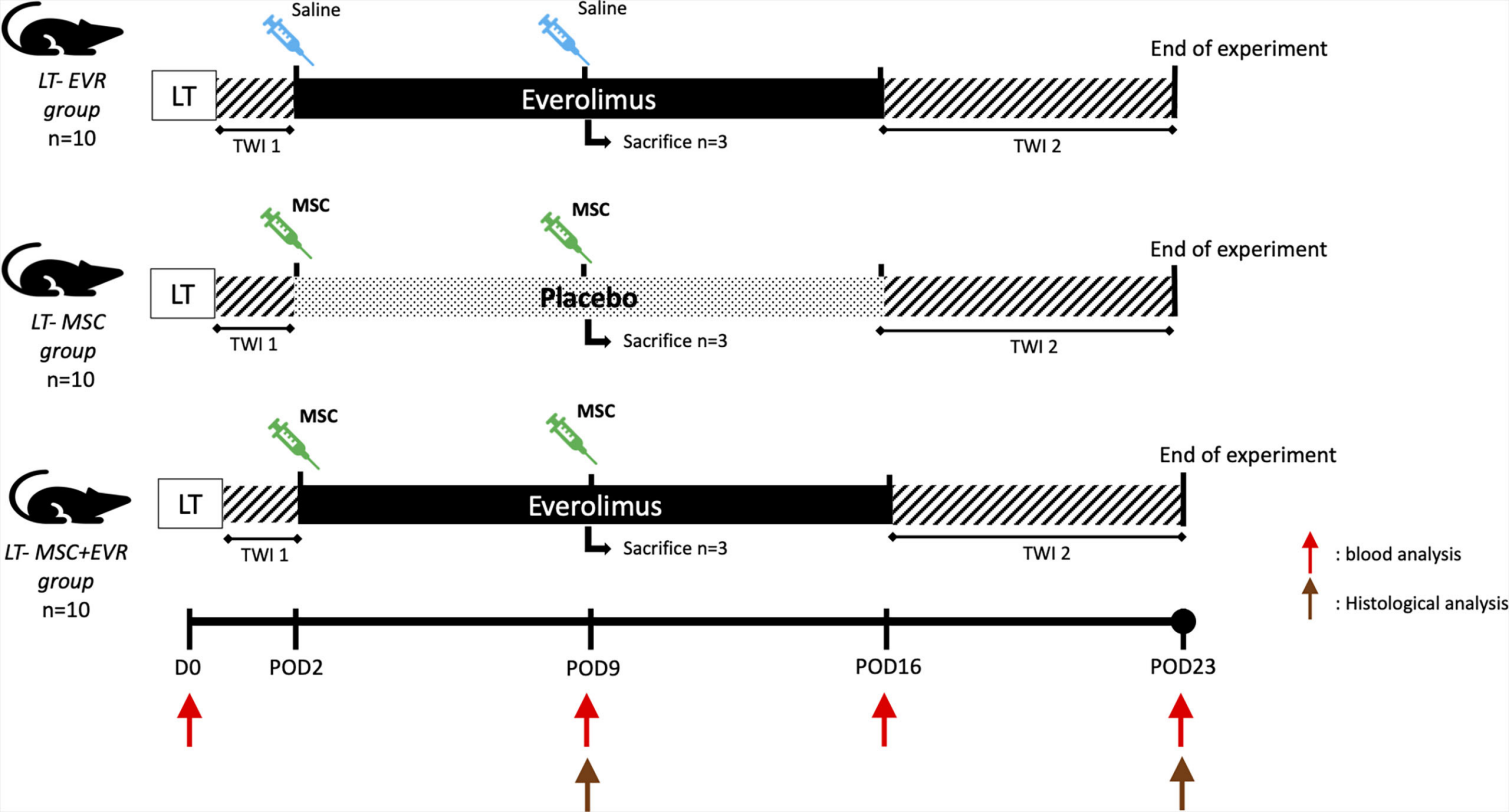
Evaluation of the association of MSCs and everolimus in a liver transplantation model (experimental design). (EVR, everolimus; LT, liver transplantation; MSC, mesenchymal stromal cell; POD, post-operative day; TWI, time without immunosuppression -1: first post-operative 48h, -2: from POD16 to POD23 (end of experiment); Red arrow, blood analysis; Brown arrow, histological analysis).

In a second part, the association of MSCs and everolimus was tested in a rat LT model. Accordingly, 10-week-old Dark Agouti male rats and 8-week-old Lewis male rats were used as liver donors and recipients, respectively. A non-arterialized orthotopic LT model in a rat was established using a modified Kamada’s two-cuff technique with adapted 3D-printed instruments and cuffs generated with a *Form2* 3-D printer (Formlabs, MA, USA) (22, 23). Directly following surgery, the rats were kept during 1 hour in an intensive care unit (Vetario, UK) with an O_2_-, temperature- and humidity- controlled environment. 48 hours after LT the rats were randomized in one of the three experimental groups (**Figure 2**) as follows:

• *LT-EVR group* (n=10): sc everolimus (0.25mg/kg/day) from post-operative day (POD) 2 to POD16 and iv saline injection (1ml through the penile vein) at POD2 and POD9.
• *LT-MSC group* (n=10): sc placebo from POD2 to POD16 and iv MSC injection (3.10^6^ cells/kg) at POD2 and POD9.
• *LT-MSC+EVR* group (n=10): sc everolimus from POD2 to POD16 and iv MSC injection (3.10^6^ cells/kg) at POD 2 and POD9.

### 2.5 Liver and Renal Function Measurements and Histological Analysis

In each LT-group (**Figure 2**), blood samples were taken through the rat tail vein at 4 time points: just before LT (D0), POD9, 16 and 23, in order to measure the serum levels of creatinine, alanine aminotransferase (ALT), aminotransferase (AST) and total bilirubin.

At POD 9, 3 rats from each group were sacrificed for liver histological analysis. At POD23 the living remaining rats of each group were sacrificed for laboratory tests and histological analysis. Liver tissue was obtained for pathological examination after animal sacrifice (**Figure 2**). To determine Banff score and fibrosis score (METAVIR), samples were fixed in 10% formalin, embedded in paraffin, sliced into 5µm-thick sections and stained with Hematoxylin and Eosin (H&E) or Masson’s trichrome. A liver pathologist (N.B.) blindly examined the H&E-stained slices and trichrome-stained slices for Banff score and for fibrosis score (METAVIR) grading, respectively (24). To visualize FoxP3 positive cells in the graft, liver samples were fixed in paraformaldehyde 4% for 24 hours and then embedded in paraffin. Sections (5µm) were dewaxed and then gradually hydrated before immunohistochemistry and were subjected to antigen retrieval EDTA buffer (Dako, #S2367). Endogenous peroxidase activity was blocked with 3% hydrogen peroxide (Merck 30%, #107209) for 20 min at RT. Non-specific binding was constrained by incubation for 10min with protein block reagent (Cell Signaling Technology, #15019L). Then, sections were incubated for 60min at room temperature with primary antibodies: Rabbit anti FoxP3 (ABCAM# 215206 1/250 in diluent Dako#S3022). After washing, sections were incubated for 30 min with secondary antibodies (Envision anti-Rabbit/HRP, Dako #K4003), and subsequently washed and exposed to horseradish peroxidase for 30min. Immunoreactivity was detected using 3,3′-Diaminobenzidine (Dako #K3468). N.B. blindly counted the number of FoxP3+ cells at 40x magnification in three high power fields from the graft sections.

### 2.6 Treg Flow Cytometry Analysis

Cells were harvested from whole blood samples and FACS was performed using 3 fluorochrome-conjugated antibodies: i) fluorescein-conjugated anti-CD4 (R&D, #967211) ii) Rα PE-conjugated anti-CD25 (R&D, #968436) iii) Alexa Fluor-conjugated anti-FoxP3 (R&D, #968435). Beforehand, a red blood cells lysis buffer (eBioscience, #00433357) was used to remove Red Blood cells from the whole blood samples. A FoxP3/transcription factor permeabilization buffer (R&D, #43481) was used for nuclear permeabilization before incubation with anti-FoxP3 antibody. Flow cytometry was performed on an FACS Canto II flow cytometer to evaluate cell fluorescence. Obtained data were analyzed using FACS Diva software (BD, San Jose, CA). Percentages of FoxP3+/CD4+CD25+ cells and of FoxP3+/CD4+ total cells were calculated. Fluorescence Minus One Control was defined for FoxP3 using an isotype control. Gating strategy is given in **Supplementary Figure 2**.

### 2.7 Statistical Analysis

Data are presented as median [P25-P75]. GraphPad Prism version 7.00 Software (San Diego, CA) was used for statistical analysis. A *p-*value <0.05 was considered as statistically significant. Kruskal-Wallis test, Mann-Whitney test or Wilcoxon test was used when appropriate. Graft survival was analyzed with the Kaplan-Meier method using the log-rank (Mantel-Cox) test.

## 3 Results

### 3.1 Everolimus Can be Efficiently Delivered in Rats *via* a Subcutaneous Osmotic Pump

**Figure 3:**
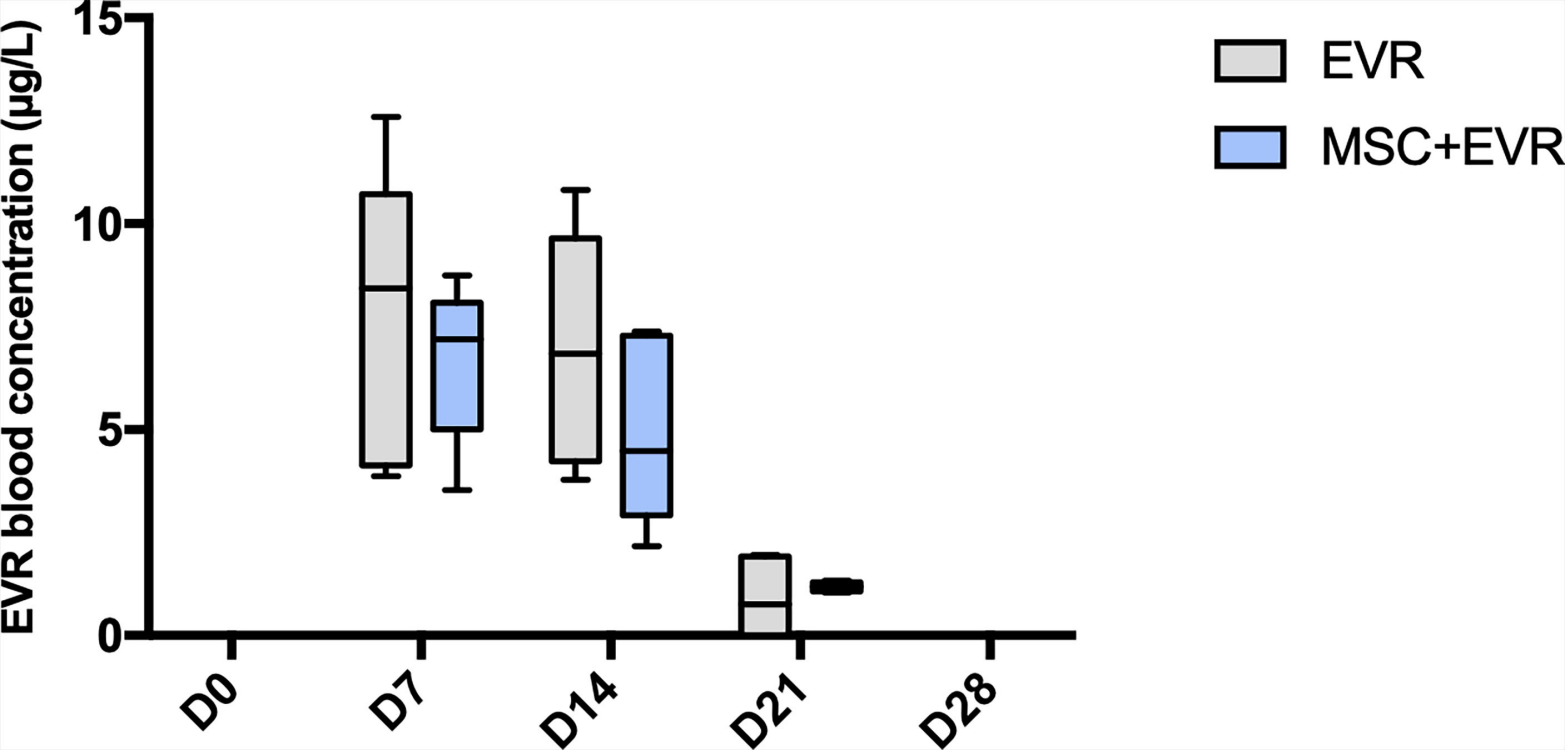
Evolution of everolimus blood concentration. In the 2 groups (n = 6/group) receiving everolimus with osmotic pumps, the drug could be efficiently delivered with a statistically increased concentration during 14 days. Plots display the median (25th and 75th percentiles) of the distributions (boxes), and whiskers extend to the minimal and maximal values. There was no statistical difference between the groups at any time point (Mann-Whitney test). (D, Day; EVR, Everolimus ;MSC, mesenchymal stromal cells).

In the *EVR group a*nd *MSC+EVR group*, everolimus was efficiently delivered in all rats (with no significant difference between these groups) (**Figure 3**). With a sc delivery of 0.25mg/kg/day, the concentration of everolimus measured in the blood at D7 was 8.43µg/L [4.13-10.73] in the *Evero group* and 7.19 µg/L [5.01-8.08] in the *MSC+Evero group* (P= 0.39). At D14 it was 6.84 µg/L [4.24-9.64] and 4.49 µg/L [2.92-7.28] in the *Evero group* and in the *MSC+Evero group*, respectively (*P=* 0.24). At D21 the blood concentration of everolimus was close to 0 µg/L in all rats (*Evero group*: 0.76 µg/L [0-1.95]; *MSC+Evero group*: 1.18 µg/L [1.07-1.29]; *P* >.99) and at 0 µg/L in all rats at D28. Blood levels of everolimus were undetectable at all timepoints in the placebo group.

### 3.2 MSC-Induced Treg Expansion Is Not Inhibited by Everolimus Adjunction

**Figure 4:**
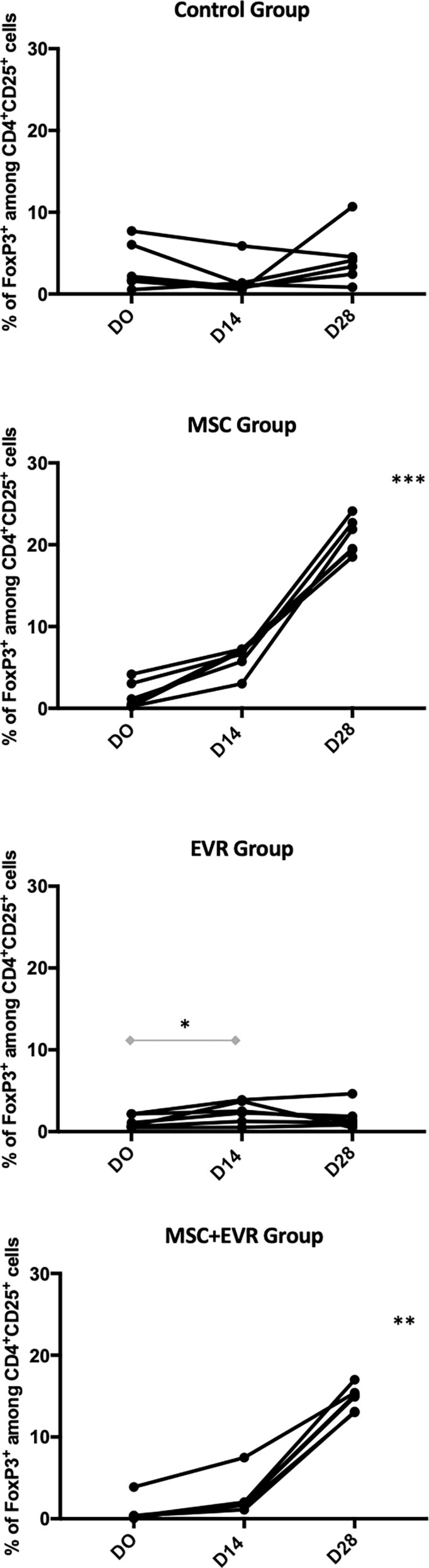
Evolution of CD4^+^ CD25^+^ FoxP3^+^ Treg percentage. Evolution of the FoxP3^+^ cells among CD4^+^CD25^+^ percentages in each group (each line represents a rat). Rats of the *Control group* kept a stable percentage of circulating Tregs until D28 (insignificant, NS, *P=0.43*) as well as *EVR group* (*P=0.052*). An important and significant expansion of FoxP3 percentage until D28 was observed in the rats of both the *MSC group* (***, *P*= 0.0001) and of the *MSC+EVR group* (**, *P*= 0.0017). The Friedman test was used for statistical analysis. Of note, when compared to D0, a slight but significant increase in the FoxP3+ Tregs percentage was observed in the *EVR group* at D14 (*, *P=0.03, Wilcoxon test*). (D: Day; EVR: Everolimus; MSC: mesenchymal stromal cells).

The percentage of *CD4^+^ CD25^+^* FoxP3^+^ Tregs amongst CD4^+^ CD25^+^ cells measured in each group was not statistically different in the 4 groups at D0 with 1.97% [1.33-6.47] in the *Control group*, 0.84% [0.24-3.33] in the *MSC group*, 0.89% [0.58-2.16] in the *Evero group* and 0.34% [0.24-1.26] in the *MSC+Evero group*; (*p=0.08*). In the *Control group*, the percentage of FoxP3+ Tregs at D14 (1.01% [0.68-2.49]) and D28 (3.74%[2.03-6.07]) remained stable without statistical difference between these timepoints. In both the *MSC Group* and the *MSC+Evero Group*, the percentage of FoxP3+ Tregs had significantly increased by D14 from their base rate (6.79% [5.06-7.21] and 1.91% [1.52-3.39] respectively) and this increase was even greater at D28 in both groups (15%[13.1-15.8] and 20% [19.2-23.1] respectively). In the *Evero Group*, FoxP3+ Treg percentage showed a slight but significant increase at D14 (2.41% [1.08-3.75] but not at D28 (1.25% [0.76-2.58]) (**Figure 4**).

### 3.3 MSCs Alone Are Not Effective in Preventing Acute Rejection

#### 3.3.1 Survival Time of Transplant Recipients

Thirty liver-transplanted rats were randomized in 3 groups (n=10/group) on POD2 (**Figure 2**). The weight of rat donors and recipients, operative- and clamping-times in addition to total ischemia time were comparable in the three groups (**Supplementary Table 1**).

**Figure 5:**
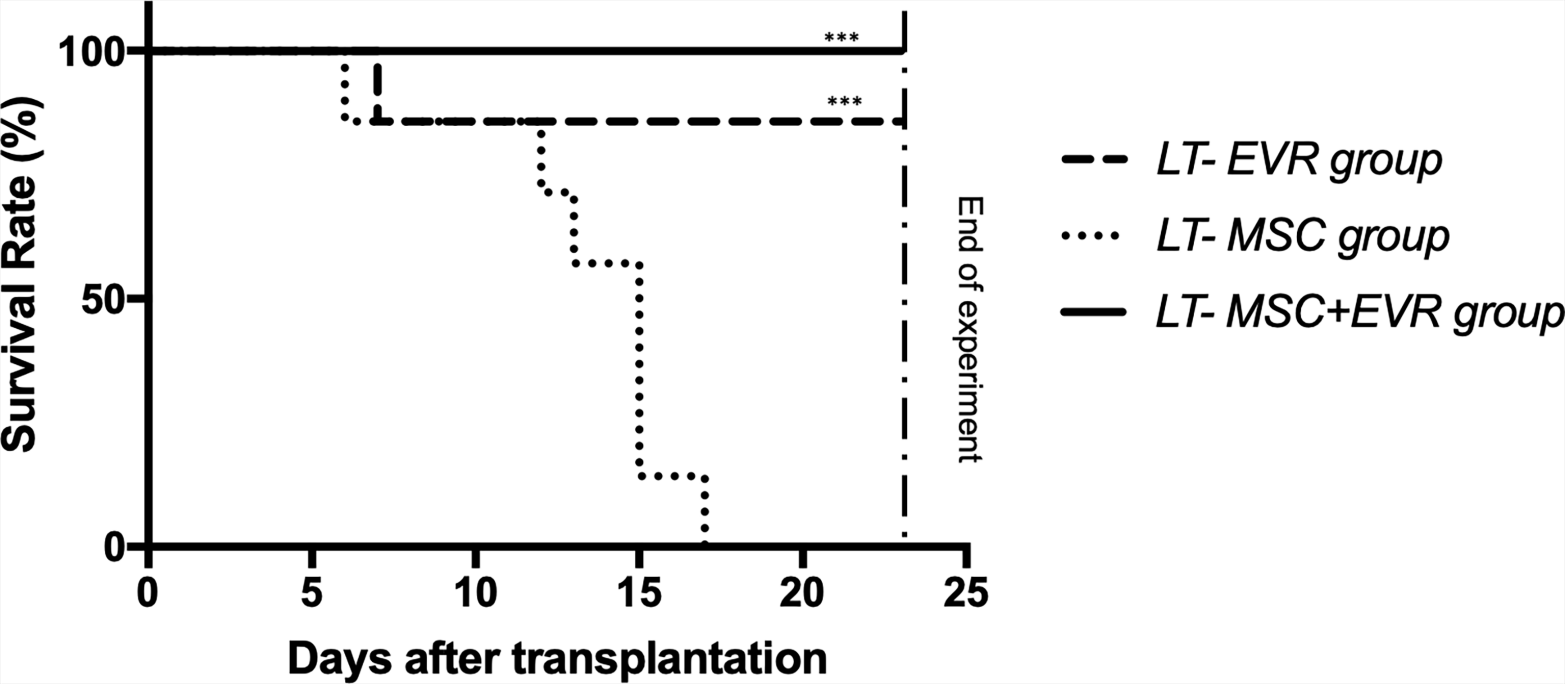
The Kaplan-Meier survival curve of liver transplant recipients. Kaplan-Meier survival curves for the LT-EVR, LT-MSC and LT-MSC+EVR groups. The survival rates for the *LT-EVR* and *LT-MSC+EVR groups* were significantly higher than those of the *LT-MSC group*. All rats of the *LT-MSC group* died between post-operative day 6 and 17; Graft survival was analyzed with the Kaplan-Meier method using the log-rank (Mantel-Cox) test. (***P < 0.001; LT, liver transplanted rat; EVR, Everolimus; MSC, mesenchymal stromal cells).

As presented in **Figure 5**, 100% and 86% of the rats of the *LT-MSC+EVR* and *LT-EVR groups*, respectively, were alive and euthanized at POD23 (after exclusion of the rats sacrificed on POD9). Rats treated with MSCs alone (*LT-MSC group*) showed a high mortality rate, with no surviving rats at the end of the experiment, and with a median survival of 15 days [12-15] (*P<*0.001).

#### 3.3.2 Liver Tests and Histological Observations of Transplanted Rats

**Figure 6:**
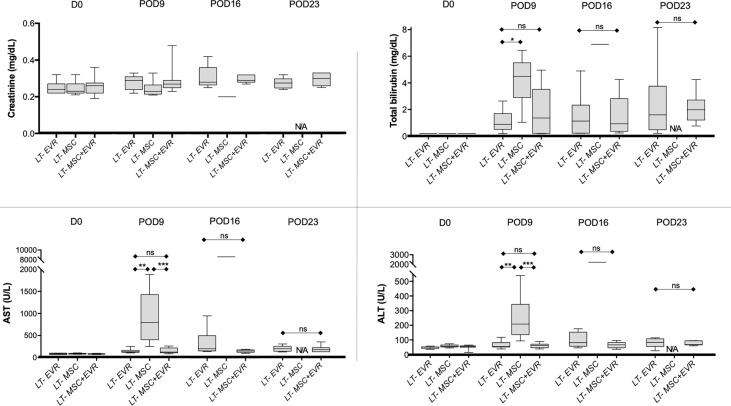
Levels of creatinine, total bilirubin, aspartate aminotransferase (AST) and alanine aminotransferase (ALT) in the blood at D0, post-operative 9, 16 and 23 in recipients after liver transplantation. Plots display the median (25th and 75th percentiles) of the distributions (boxes), and whiskers extend to the minimal and maximal values. The Kruskal-Wallis test followed by Dunn’s multiple was used when comparing three groups, and the Mann-Whitney test was carried out when comparing just two groups at a timepoint. At D0 n = 10 per group, at D9 n = 9/9/10, at D16 n = 9/1/10 and at D23 n = 9/0/10 for *LT-EVR/LT-MSC/LT-MSC+EVR groups*, respectively. (*P < 0.05, **P < 0.01 ***P < 0.001; ns, P > 0.05; ALT, alanine aminotransferase; AST, aminotransferase; D, Day; EVR, Everolimus; MSC, mesenchymal stromal cells; POD, post-operative day).

Pre-operative laboratory kidney and liver tests were similar in the three LT-groups (**Figure 6**). At POD9, rats from the *LT-MSC group* showed signs of acute rejection with major cytolysis: AST and ALT levels of 793 U/L [403.5-1425] and 210 U/L [138-343.5], respectively (**Figure 6**). Rats from the *LT-EVR group* and *LT- MSC+EVR group* had only slightly increased liver test results with AST levels of 130 U/L [118-164] and 126 U/L [109.5-219], respectively, and ALT levels at 58 U/L [55-84.5] and 65 U/L [50-74.5], respectively (**Figure 6**). On POD16 only one rat from the *LT-MSC* group remained alive for biological analysis and revealed extremely high cytolysis (AST and ALT at 8702 and 2162 U/L, respectively). AST and ALT levels were slightly increased in the *LT-EVR* and *LT- MSC+EVR* groups on POD 16 (AST: 206 U/L [156.5-504] and 155U/L [126-180] respectively, P=0.06; ALT: 83 U/L [58.2-153.5] and 68 U/L [48-84] respectively, P= 0.29) and on POD 23 (AST: 191.5 U/L [134.8-247.5] and 170 U/L [123-217] respectively, P=0.8; ALT: 86 U/L [57.2-110.3] and 71 U/L [68-97] respectively, P= 0.73) without any significant differences between these groups. On POD23 no rat remained alive in *the LT- MSC group*.

**Figure 7:**
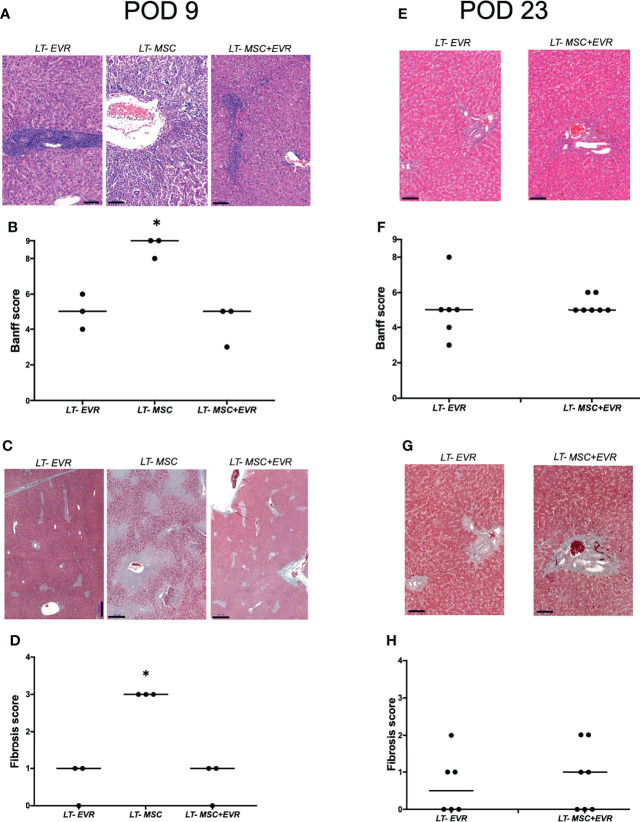
Histological analysis.**(A)** H&E staining of liver graft and, **(B)** histological grading of liver graft acute rejection severity using Banff schema at D9 post-LT; **(C)** Masson’s trichrome staining highlighting liver graft fibrosis lesions **(D)** Histological grading of liver graft fibrosis severity with METAVIR score at D9 post-LT; **(E)** H&E staining of liver graft and, **(F)** histological grading of the severity of acute liver graft rejection using Banff schema at D23 post-LT; **(G)** Masson’s trichrome staining highlighting liver graft fibrosis lesions and **(D)** histological grading of liver graft fibrosis severity with METAVIR score at D23 post-LT. [*P < 0.05; **(B, D)** Kruskal-Wallis test; **(F, H)** Mann Whitney test; scale bars = 100 µM **(A, E, G)**, 500µM **(C)**].

**Figure 8:**
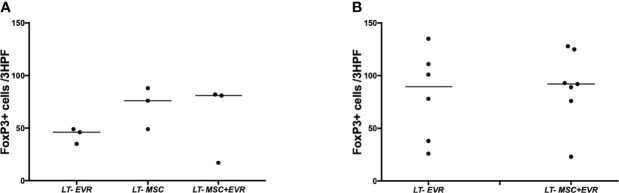
Treg infiltration in the liver graft. Number of FoxP3+ cells at 40x magnification (per three high power fields) by examining graft section of each group after immunohistochemistry on POD 9 **(A)** and at the end of the experiment **(B)**.

Three rats from each group were sacrificed on POD9 in addition to all the living rats of each group on POD23, in order to evaluate the severity of acute rejection and fibrosis (**Figure 7**) as well as the presence of Treg cells in liver allografts (**Figure 8**). On POD9 the rats in the groups receiving everolimus (*LT-EVR* and *LT- MSC+EVR* groups) showed histological signs of moderate acute rejection with both a median Banff score of 5/9 (**Figures 7A, B**) and mild fibrosis (F1) (**Figures 7C, D**), while conversely, those treated with MSCs alone (*LT-MSC group*) showed histological signs of severe acute rejection with a median Banff score of 9/9 (**Figures 7A, B**) and severe fibrosis (F3) (**Figures 7C, D**). At POD23 the median Banff score was similar in the sacrificed rats of the *LT-EVR* (n=6) and *LT-MSC+EVR* (n=6) groups (5/9) (**Figures 7E, F**). Liver fibrosis was also similar, with a median lower than F1 in both groups (**Figures 7G, H**). On POD9, rats receiving MSC (LT-MSC and LT MSC+EVR groups) seemed to have a higher, but not statistically significant, count of FoxP3+ cells in the graft (76 [35-49] and 81 [17-82] FoxP3+ cells/3HPF respectively) when compared to rats of the LT-EVR group (46 [35-49] FoxP3+ cells/3HPF) (**Figure 8A**). At POD23 the number of Tregs in the liver graft was comparable in the LT-EVR (89.5 [35-117] FoxP3+ cells/3HPF) and in the LT-MSC+EVR groups (92 [76-128] FoxP3+ cells/3HPF) (P>0.99) (**Figure 8B**).

## 4 Discussion

MSCs, thanks to their immunomodulatory properties including their positive effect on Treg cells, have given rise to a therapy that is a promising approach in the field of SOT (6). In this study we confirmed that MSC infusion promotes Treg expansion in rats, and we additionally showed that everolimus might have a transient positive impact *per se* on the circulating Treg percentage. More interestingly, we found that everolimus has no negative impact on Treg expansion when combined with MSCs. Nevertheless, in a LT model in the rat we observed that MSC injections alone were not effective in preventing acute rejection, and additionally that combining MSCs and everolimus failed to show any synergistic effect when compared to everolimus alone.

Our team has recently reported on two phase I-II clinical trials investigating the effect of MSCs in kidney and liver transplantations (11, 16, 25). In these two studies, and in other reported clinical trials, the effect of MSCs as an immunosuppressive therapy are encouraging but the results remain inconstant and thus not yet clinically relevant (6). A predominant role among the various factors incriminated in this inconstancy may be the alteration of MSC and Treg functions due to the associated ISDs. In this perspective, it is important to note that the majority of clinical trials investigating the effect of MSCs in SOT have used a concomitant “classical immunosuppressive regimen” with CNIs as the central ISDs (4). The dilemma that has emerged relates to the probable CNI toxicity against Treg proliferation and function. Interestingly, it has been reported in an animal model that Tregs injected after LT could induce tolerance (26), and many clinical trials studying Treg injections after SOT have shown encouraging data pointing to the same conclusion - even if these studies were focused on safety (9). It therefore seems logical to try to reduce the ISDs that could impair the immunoregulatory functions of Tregs. Furthermore, CNIs may impair MSC functions (12–15). Therefore, MSC-based therapy requires additional pre-clinical investigations to define the best synergistic “MSC-compatible” ISDs. Meanwhile, mTOR inhibitors are an alternative ISD regularly used after SOT to replace or minimize CNIs, especially in cases of malignancies or of CNI-related renal toxicity (27). In contrast to what has been observed with CNIs, mTOR inhibitors have been shown to have a positive effect on Treg function/proliferation in pre-clinical and clinical studies (14, 15, 28, 29) including in LT (30). Nevertheless, some *in vitro* data have suggested that rapamycin could be deleterious for MSC proliferation and immunomodulatory properties, while *in vivo* MSCs and rapamycin synergize to promote graft tolerance in allograft rejection models (19, 20).

In this work, our first aim was to observe the effect of a single dose of MSCs with EVR on the percentage of Tregs in blood and subsequently weigh up the effectiveness of this association in preventing acute rejection in a LT model.

Firstly, we were able to demonstrate that EVR, despite its very poor solubility in water, could be efficiently and reproducibly delivered through a sc osmotic pump to rats. As EVR is metabolized by the liver, levels of EVR in the blood of LT rats are probably different than those of non-LT rats. However, the specific purpose of the first part of this work was to confirm the effectiveness of EVR delivery with osmotic pumps, not to compare pharmacokinetics in these two populations, which would have meant adding blood sampling in liver transplanted rats.

Our study further confirmed that MSCs significantly increase circulating CD4^+^CD25^+^FoxP3^+^ Treg percentage in Lewis rats. This effect was observed 5 days after the injection and persisted for more than 2 weeks thereafter. This increment in Treg percentage after MSC administration has already been documented in other pre-clinical studies (6, 31). In addition, rats from the *MSC+Evero group* showed a comparable increase in Treg percentage. Concurrently, everolimus alone had a positive impact on Treg expansion by 14 days. This finding suggests that everolimus may have a beneficial effect on Treg proliferation, but only while it is being administered, since the percentage of circulating Tregs returned to baseline at D28. Gedaly et al. have recently shown that mTOR inhibitors could be used to expand *ex vivo* functionally competent Tregs for clinical use (32). On the same subject, in clinical kidney transplantation it has been observed that recipients under rapamycin maintenance have increased circulating Treg levels compared to patients receiving cyclosporine (29). Another team recently confirmed these observations in LT recipients who showed increased Treg levels after being switched from tacrolimus to mTOR inhibitors (30). Additionally, a recent paper reported the results of a phase Ib trial studying the effect of 2 MSC infusions 6 months after kidney transplantation followed by a lowering of tacrolimus in combination with everolimus and prednisolone, but no effect on Treg levels or function could be found in this study (33). The mechanisms through which everolimus interacts with MSCs and Tregs was not investigated in this study. Importantly, it is not possible in this study to identify whether the MSC or everolimus are causing expansion of Tregs or deletion of other T cells as we evaluated Treg percentages rather than absolute number. Of note, it was shown that rapamycin does not directly promote Treg expansion, and that the positive effects of mTOR inhibitors on Tregs was probably due to selective inhibition having a greater action on conventional T-cells rather than on Tregs. This consequently leads to a “Treg-favoring” effect with an increase in circulating Tregs (14). Nonetheless, the different mechanisms for MSC-mediated effects on Tregs are likely to be complex and remain incompletely understood (6).

Based on these primary findings, we then tested the association of MSCs with everolimus in a LT model of acute rejection. Insufficient data are available in pre-clinical transplantation models studying the association of mTOR and MSCs. Studies have been carried out in heart (19) and in pancreas islet (20) transplantation models but none in a LT model. In this study we used a well-described model of acute rejection of liver graft in rats using Dark Agouti as donors and Lewis rats as recipients. Circulating levels of Treg in liver transplant recipient were not assessed in the present study. Nevertheless, in another pre-clinical study in rats, Wang et al. showed that peripheral Treg percentages were significantly higher in liver transplant recipient after MSC injections when compared to recipient not injected with MSC. Although not significant, we observed that the number of Tregs in the grafts at 7 days after the first injection tended to be greater in those of rats receiving MSCs, but this was not confirmed at the end of the experiment. The evaluation of the circulating and intragraft levels of other immune cells subsets known to play an important role in MSC-mediated immunoregulatory properties (such as NK cells, CD8+ T cells, B cells, etc.) would have probably been interesting in the present study. Furthermore, two post-operative MSC injections in the *LT-MSC group* (without ISDs) were not an effective treatment to prevent severe acute rejection, and in this group all the rats died prematurely in comparison to rats receiving everolimus. In contrast to our results, some other studies using the same LT model have shown that MSCs alone could significantly prolong graft survival, and prevent rejection when compared to the survival of recipients not receiving any treatment (34, 35). In these studies injected MSC doses were either higher (36) or lower (34) than in our protocol, but more relevantly, MSCs were injected through the portal vein during the transplantation procedure whereas we injected cells intravenously through the penile vein on POD2 and 9. This highlights the fact that the timing and site of the injection are probably key points in achieving MSC efficiency, and that injection closer to the time of transplantation and of ischemia-reperfusion injuries is perhaps needed in order to obtain superior immunomodulatory effects.

On the other hand, liver transplanted rats treated with everolimus alone, or with everolimus in combination with MSC injections, had significantly improved survival rates and less biological or histological damage than the rats of the *LT-MSC group*. No clinical, biological of histological difference was found between these two groups and thus no effective beneficial effect of MSC adjunction with everolimus in our model. Interestingly, levels of acute rejection were stable even 7 days after everolimus withdrawal. In 2018, in a model of islet allograft in mice, Duan et al. also found, that treatment with MSCs alone or with low-dose mTOR inhibitors was not effective in treating acute rejection and prolonging graft survival, but that the combination of MSCs and low-dose rapamycin could significantly increase graft function and survival (20). Nevertheless, in that experiment the survival of mice treated only with rapamycin was similar to those treated with MSCs alone, while in our study everolimus alone was effective in significantly lessening acute rejection when compared to MSCs alone. This might suggest that our “low dose everolimus protocol” led to a sufficient concentration of everolimus to alleviate acute rejection and thus conceal the potential beneficial effect of MSC adjunction. An additional group studied the combination of rapamycin (2mg/kg/d from POD 0 to 13) with MSCs (1 iv injection of 1x10^6^ cells at POD 1) in a heart allograft transplantation model (19) and found that MSCs alone led to a reduction of graft rejection and doubled graft survival compared to a control group. Furthermore, a combination of MSCs and rapamycin was even able to induce a tolerance of the graft, with long-term survival and normal histology.

In our LT model, MSCs alone were not effective in preventing acute rejection, and no beneficial effect of associating MSCs with everolimus was found. Nevertheless, the synergistic effect of MSCs with everolimus could potentially be revealed if the timeframe from transplantation and ISD withdrawal was extended further than in our present protocol. When comparing other LT models to our own, MSC injections were systematically performed earlier than in our case, but our choice to inject MSCs at D2 was carried out in order to reproduce our previous clinical data. Indeed, MSC injections before POD2 might be logistically difficult in a program of deceased LT (16).

In conclusion, despite a significant increase of the percentage of circulating Tregs after one injection of MSCs, MSCs were not effective in preventing severe acute rejection in our LT model. In addition, no effect of the association of MSCs with everolimus was found, when compared to everolimus alone. Everolimus alone could significantly alleviate acute rejection, showing a stable level of rejection even one week after its final administration. Furthermore, the addition of MSCs had no observable positive synergistic impact on acute rejection treatment, or prevention, when compared to everolimus alone. However, it is possible that MSC injections closer to LT time, and a longer follow-up, would reveal some differences in graft survival and tolerance induction. Given these results, the association of everolimus and MSCs in SOT deserves further investigation.

## Data Availability Statement

The raw data supporting the conclusions of this article will be made available by the authors, without undue reservation.

## Ethics Statement

The animal study was reviewed and approved by Commission Ethique Animale ULiège.

## Author Contributions

Conception and design of the work: MV, OD, and FJ. Data collection: MV and LP. Data analysis and interpretation: MV, PE, NB, OD, and FJ. Drafting the article: MV and OD. Critical revision of the article: PE and FJ. All authors contributed to the article and approved the submitted version.

## Funding

This work was supported by grants with no role in the conduct of the study. MV, PE, and FJ are Fellows of the *Fonds National de la Recherche Scientifique* (FNRS) and received support from the FNRS (Research Credit 2013/2015) and from the University of Liege (Fonds Spéciaux à la Recherche - Fondation Léon Frédéricq (ACiiRT)). This study was supported by 2 *Crédits classiques* from the University Hospital of Liege and Awards from the Fondation Léon Frédéricq, University of Liege, (2017 and 2018).

## Conflict of Interest

The authors declare that the research was conducted in the absence of any commercial or financial relationships that could be construed as a potential conflict of interest.

## Publisher’s Note

All claims expressed in this article are solely those of the authors and do not necessarily represent those of their affiliated organizations, or those of the publisher, the editors and the reviewers. Any product that may be evaluated in this article, or claim that may be made by its manufacturer, is not guaranteed or endorsed by the publisher.
