# Supplementary figures and images for: Effect of the Combination of Everolimus and Mesenchymal Stromal Cells on Regulatory T Cells Levels and in a Liver Transplant Rejection Model in Rats
Source: Front Immunol. 2022 Jun 10;13:877953. doi: 10.3389/fimmu.2022.877953 (PMC9226583; doi:10.3389/fimmu.2022.877953)

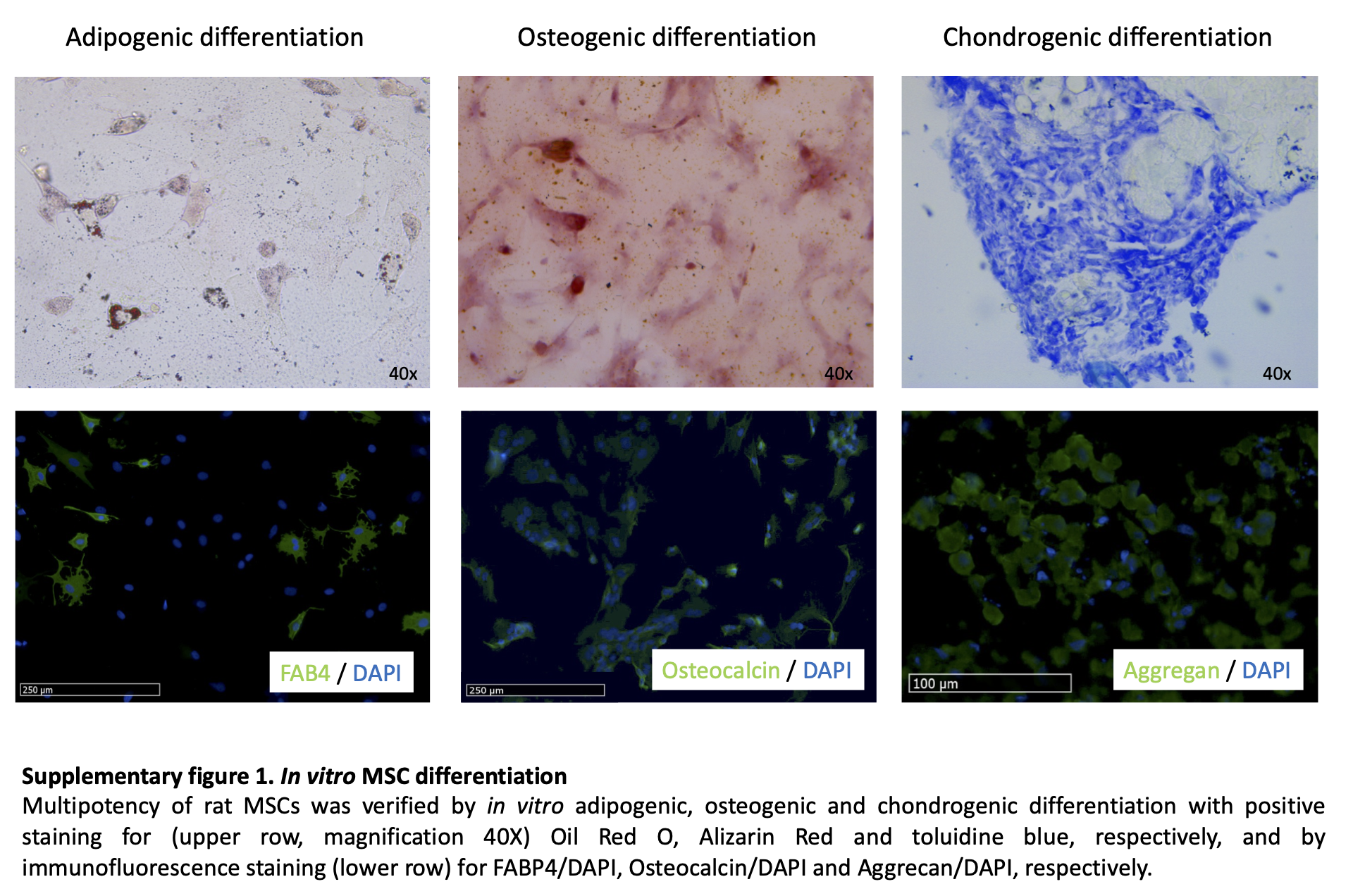

Supplement: Supplementary file 1 [file Image_1.tiff]

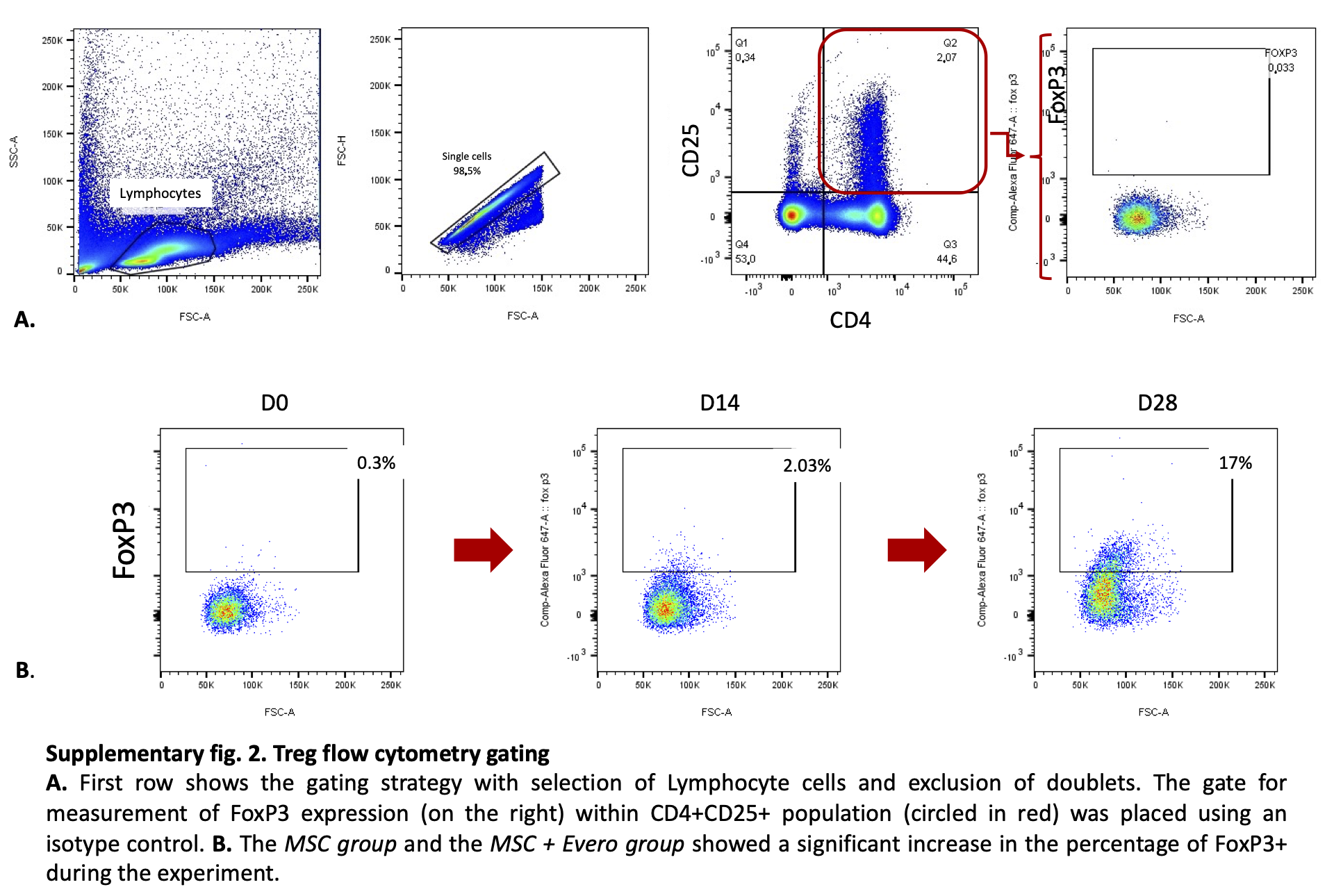

Supplement: Supplementary file 2 [file Image_2.tiff]

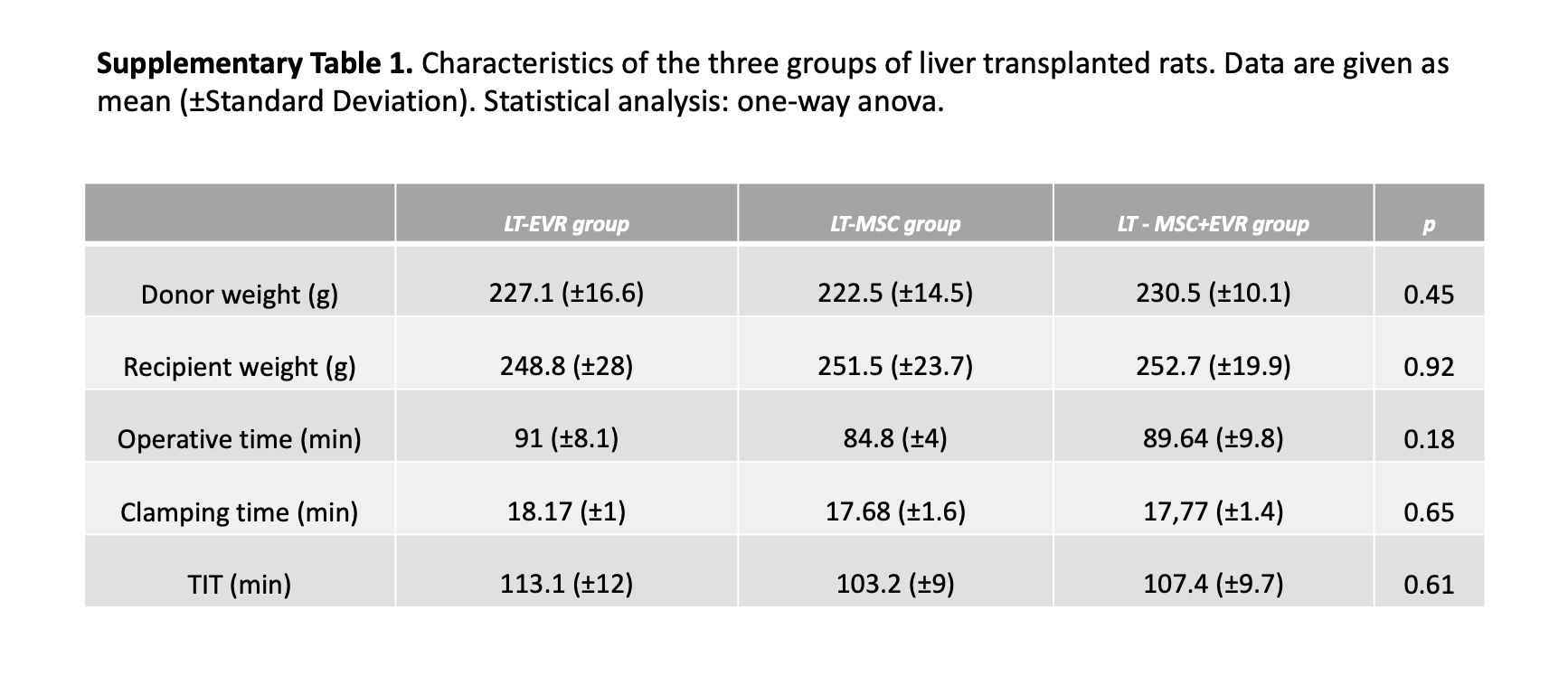

Supplement: Supplementary file 3 [file Image_3.tiff]
